# On the Oxidative Valorization of Lignin to High‐Value Chemicals: A Critical Review of Opportunities and Challenges

**DOI:** 10.1002/cssc.202201232

**Published:** 2022-09-19

**Authors:** Omar Y. Abdelaziz, Ida Clemmensen, Sebastian Meier, Carina A. E. Costa, Alírio E. Rodrigues, Christian P. Hulteberg, Anders Riisager

**Affiliations:** ^1^ Department of Chemical Engineering Lund University Naturvetarvägen 14 SE-221 00 Lund Sweden; ^2^ Department of Chemistry Technical University of Denmark Kemitorvet 207 DK-2800 Kgs. Lyngby Denmark; ^3^ Laboratory of Separation and Reaction Engineering–Laboratory of Catalysis and Materials (LSRE-LCM) Department of Chemical Engineering Faculty of Engineering University of Porto Rua Dr. Roberto Frias 4200-465 Porto Portugal; ^4^ Associate Laboratory in Chemical Engineering (ALiCE) Department of Chemical Engineering Faculty of Engineering University of Porto Rua Dr. Roberto Frias 4200-465 Porto Portugal

**Keywords:** analytical methods, biomass, heterogeneous catalysis, lignin valorization, oxidative depolymerization

## Abstract

The efficient valorization of lignin is crucial if we are to replace current petroleum‐based feedstock and establish more sustainable and competitive lignocellulosic biorefineries. Pulp and paper mills and second‐generation biorefineries produce large quantities of low‐value technical lignin as a by‐product, which is often combusted on‐site for energy recovery. This Review focuses on the conversion of technical lignins by oxidative depolymerization employing heterogeneous catalysts. It scrutinizes the current literature describing the use of various heterogeneous catalysts in the oxidative depolymerization of lignin and includes a comparison of the methods, catalyst loadings, reaction media, and types of catalyst applied, as well as the reaction products and yields. Furthermore, current techniques for the determination of product yields and product recovery are discussed. Finally, challenges and suggestions for future approaches are outlined.

## Graphical abstract

**Lignin valorization**: Technical lignin is a large‐volume and low‐cost industrial resource for making renewable aromatic chemicals. Oxidative depolymerization with heterogeneous catalysts allows viable upgrading to value‐added aromatic monomers and more sustainable biorefineries. This Review summarizes and discusses performance–reactivity patterns and product analytics and recovery for the efficient oxidative valorization of technical lignin.

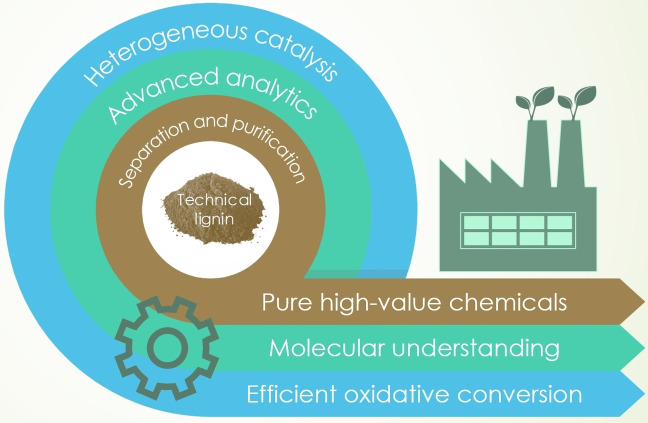

## 1 Introduction

Lignin is the most abundant naturally occurring aromatic polymer on Earth and has excellent potential as a renewable feedstock for the production of chemicals, fuels, and functional materials. Lignin is also a major side‐product from the pulp and paper industry and cellulosic ethanol production, presenting a bulk raw material commonly referred to as technical lignin.^[1]^ Despite its immense potential, lignin is still vastly underutilized and is primarily burned for energy recovery. The annual global chemical (Kraft) pulp production has been estimated to be 130 million tons, and about 50 million tons of lignin are obtained as a side‐product.[^2^, ^3^] The potential of lignin as a sustainable feedstock for various applications is derived from its high abundance in nature, making it the most abundant aromatic compound in nature. The valorization of lignin has the potential to improve both the economic viability and the environmental performance of forest‐based industries.^[4]^

The development of successful strategies for lignin valorization necessitates efficient technologies for lignin depolymerization. Lignin depolymerization strategies reported in the literature involve acid‐catalyzed,^[5]^ base‐catalyzed,^[6]^ biochemical,^[7]^ oxidative,^[8]^ reductive,^[9]^ and thermal^[10]^ methods. Each method is associated with its own benefits and drawbacks, and complex product mixtures are usually obtained.[^3^, ^4^] The variations in operating conditions between the different depolymerization methods can also impact the compositions and yields of the resulting products significantly. Moreover, each kind of technical lignin is unique with regard to its purity, dispersity, molecular weight, and chemical structure.[^11^, ^12^] Thus, selecting the appropriate depolymerization method for every lignin type is key to ensure product efficacy.

Among different depolymerization methods, oxidative depolymerization is generally attractive due to the relatively mild operating conditions and their ability to produce targeted products with multiple functionalities. Oxidative lignin depolymerization can be used to produce valuable chemicals such as aromatic aldehydes and acids, as well as aliphatic carboxylic acids.[^13^, ^14^, ^15^, ^16^] The oxidative approach has also been recommended as one of the most promising methods for the transformation of lignin into high‐value chemicals that are suitable for production on commercial scale.^[17]^ Due to its considerable potential, the oxidative depolymerization of lignin is the main subject of the present Review.

Most oxidative lignin depolymerization studies have been carried out using oxygen as the oxidizing agent in alkaline media, thus enabling the selective production of aromatic aldehydes (i. e., vanillin and syringaldehyde).[^13^, ^18^] A number of Reviews have already been published on the oxidative valorization of lignin, and the reader is referred to these Reviews for general references and detailed information on this topic.[^4^, ^8^, ^9^, ^14^, ^15^, ^19^, ^20^, ^21^, ^22^, ^23^, ^24^] Here, focus is on providing a critical Review of the challenges and opportunities in understanding the conversion of technical lignin, via heterogeneous catalysis under oxidative conditions, on the molecular level. The different heterogeneous catalyst systems are summarized and discussed in relation to the type of lignin, the operating conditions used, and the resulting products. Aspects associated with this kind of conversion, including methods for product analysis, separation, and purification, are also surveyed. Thus, the present Review is complementary to existing literature, providing important insights into parameters and methodologies especially relevant for the oxidative valorization of technical lignin.

## 2 Lignin Composition and Structure

### 2.1 Monolignols

**Figure 1:**
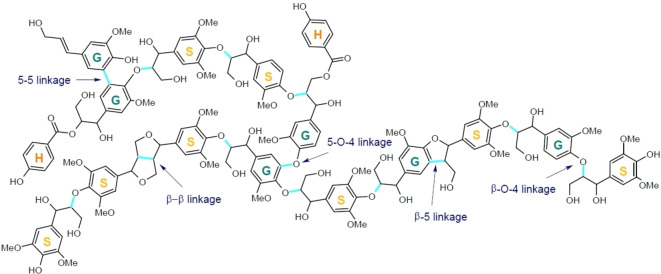
Model of the polymer structure of lignin, showing its general features and the main types of linkage. Adapted from Ref. [25], under the terms of the Creative Commons Attribution 4.0 International License (http://creativecommons.org/licenses/by/4.0/).

Lignin contains a mixture of aromatic and aliphatic moieties and forms the most complex structure among the naturally occurring polymers. The structure consists of a three‐dimensional network with different phenylpropanoid units connected by ether (C−O) or carbon–carbon (C−C) linkages in a random distribution (Figure 1). Lignin is covalently linked to hemicelluloses, cross‐links plant polysaccharides, and confers hydrophobicity on materials containing it. The highly branched structure makes lignin‐containing materials rigid.

**Figure 2:**
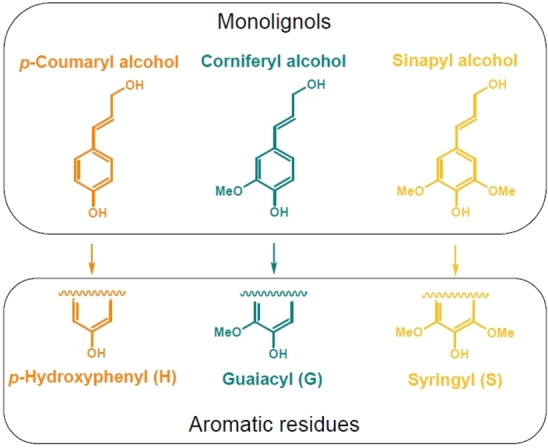
Chemical structures of the monolignols *p*‐coumaryl alcohol, coniferyl alcohol, and sinapyl alcohol, and the corresponding aromatic residues they form in lignin *p*‐hydroxyphenyl (H), guaiacyl (G), and syringyl (S).

Lignin is produced in plants through biosynthesis by the oxidative free‐radical polymerization of the three *p*‐hydroxycinnamyl alcohol monomers called monolignols: *p*‐coumaryl alcohol, coniferyl alcohol, and sinapyl alcohol.[^25^, ^26^] The monomers are phenylpropanoids with different numbers of methoxy groups attached to the aromatic ring. They correspond to the three phenolic moieties formed in lignin, namely *p*‐hydroxyphenyl (H), guaiacyl (G), and syringyl (S). The monolignols and corresponding aromatic residues are depicted in Figure 2.

The contents of the H, G, and S units vary depending on the source of the lignocellulosic biomass, and lignin can be broadly differentiated by plant origin (i. e., softwood, hardwood, or non‐wood sources). Softwood lignin, also known as guaiacyl lignin, consists almost exclusively of G units, and, in some cases, a small amount of H units. Hardwood lignin, which is also known as syringyl‐guaiacyl lignin, contains a mixture of G and S units. The ratio varies from an approximately equal percentage of the two units to three times higher levels of syringyl. Some hardwood lignin also contains a small fraction of H units. The last category of lignins is grass lignin, also known as *p*‐hydroxyphenyl‐, guaiacyl‐, syringyl‐lignin. This type of lignin contains all three units, and therefore includes a higher proportion of H units than other kinds of lignin.^[27]^ Table 1 summarizes the approximate distribution of the monolignols in the three different types of biomass.

**Table 1:** Distribution of H, G, and S units in lignin from different types of biomass.[^27^, ^28^]

Biomass type	Monolignol fraction [%]		
	*p*‐Coumaryl alcohol	Coniferyl alcohol	Sinapyl alcohol
softwood	<5	>95	0^[a]^
hardwood	0–8	25–50	45–75
grasses	5–35	33–80	20–55

[a] With some exceptions.

### 2.2 Interunit linkages

**Figure 3:**
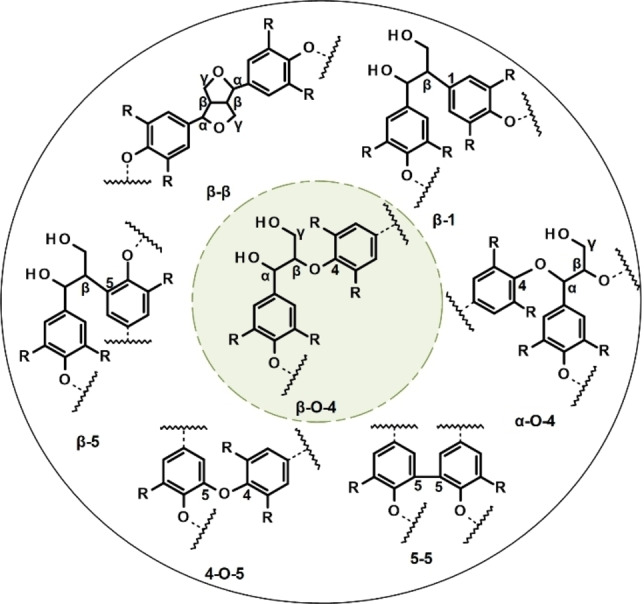
Main interunit linkages formed in the lignin biopolymer. Adapted from Ref. [30].

Different ether and carbon–carbon linkages connect the monomers in lignin with an apparently random distribution. The polymerization of monolignols proceeds by the oxidative radicalization of the phenolic hydroxyl groups and subsequent radical coupling. The resulting phenolic radical is stabilized through delocalization of its unpaired electron at the 1, 5, and/or β position, restricted by the substituted groups present on the aromatic ring. Coupling reactions at any of these positions result in polymers linked by C−O bonds (β‐O‐4, α‐O‐4, and 4‐O‐5) and C−C bonds (β‐β, β‐5, β‐1, and 5–5). The arylglycerol‐β‐aryl ether (β‐O‐4) is the most well‐known interunit linkage and is by far the most abundant and the easiest to break, which is a key aspect in lignin depolymerization.[^29^, ^30^] Some of the most common types of linkage are shown in Figure 3, and their abundance and functional groups in softwood, hardwood, and grass lignins, together with their relative proportions, are listed in Table 2.

**Table 2:** Relative proportions of the main interunit linkages and functional groups found in softwood, hardwood, and grass lignins.[^31^, ^32^]

Linkage type	Proportion of linkage or functional group [%]		
	Softwood	Hardwood	Grass
β‐O‐4	45–50	50–65	74–84
α‐O‐4	2–8	7	n.d.
4‐O‐5	4–8	7–9	n.d.
β‐β	2–6	3–12	1–7
β‐5	9–12	3–11	5–11
β‐1	7–10	1–7	n.d.
5‐5	10–27	3–9	n.d.
**Functional group abundance per 100 C_9_ units**			
aliphatic hydroxy	115–120	88–166	
methoxy	90–97	139–158	
phenolic hydroxy	15–30	10–15	
carbonyl	10–20	17–24	

The distribution of monolignols in the native (proto)‐lignin (i. e., the lignins occurring in the plant cell walls) will have a considerable influence on the fraction of interunit C−C bonds.^[33]^ S units are methoxy‐substituted at the 5‐position, which hinders the formation of 5–5 and β‐5 bonds. This means that a lignin substrate containing more S units accommodates fewer C−C bonds. As a result, hardwood lignin contains the lowest number of C−C bonds and has a more linear structure than softwood and grass lignins.[^20^, ^29^] The number of C−C linkages in the lignin substrate will greatly influence the monomer yields obtained from lignin depolymerization as the C−C bonds are much more robust than C−O bonds.^[29]^ In general, the relative abundance of each interunit linkage depends on the biomass species, lignin type, and isolation technique.^[34]^

### 2.3 Technical lignin

**Figure 4:**
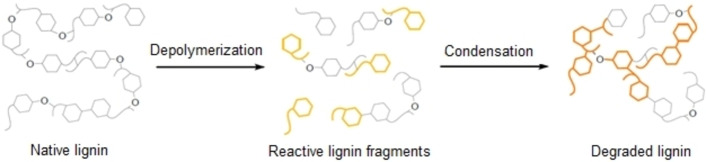
Schematic depiction of lignin degradation, where ether bonds are represented by O‐linkages. Adapted with permission from Ref. [4]; Copyright 2018, the Royal Society of Chemistry.

Technical lignins are conventionally named after the separation/extraction method employed, and include Kraft, lignosulfonate, soda, and organosolv lignins, among others. The conditions applied during lignin extraction can significantly influence the structure of the lignin, resulting in different molecular weight distributions. Furthermore, under harsh conditions, significant quantities of C−C bonds can form during extraction, in addition to their abundance in native lignin (Figure 4). This degradation of the structure makes technical lignin more resistant to depolymerization, which is why it is typically burned to recover energy.[^4^, ^29^, ^35^]

#### 2.3.1 Kraft lignin

Most of the world's paper pulp is produced by the Kraft process, which accounts for more than 80 % of the pulp production capacity.[^23^, ^36^] During the pulping process, lignin is isolated from the biomass using a solution of NaOH and Na_2_S, so‐called white liquor, at elevated temperature and pressure. This treatment cleaves the linkages to the polysaccharides and allows the dissolution of the lignin fragments. Lignin is separated from the pulp, and the solution is evaporated to obtain so‐called black liquor, which is rich in lignin. As part of the chemical recycling in the pulp mill, the black liquor is combusted and the energy is used for drying and, in the case of an energy surplus, electricity can be generated. Kraft lignin is commonly extracted from the black liquor by precipitation induced by acidification. The harsh alkaline environment to which lignin is exposed during this process leads to extensive degradation of the lignin as well as repolymerization reactions. The resulting Kraft lignin contains thiol groups and is heavily condensed with only a few β‐O‐4 bonds remaining, which can complicate its further valorization.[^4^, ^28^]

#### 2.3.2 Alkali/soda lignin

The soda process is somewhat similar to the Kraft process since it also entails the treatment of biomass with an aqueous solution of NaOH at high temperature and allows for the isolation of the lignin by lowering the pH until precipitation occurs. However, the soda process does not involve Na_2_S, which means that the reaction is less efficient than the Kraft process. The main advantage of the soda process is that the lignin obtained is free of sulfur, rendering it an attractive raw material for various applications.^[37]^

#### 2.3.3 Lignosulfonates

In sulfite pulping, lignin is extracted by breaking down the bonds to the polysaccharides using an aqueous solution called brown liquor, which contains SO_2_ and a sulfite salt (with a calcium, sodium, magnesium, or ammonium cation). Lignosulfonates are separated from the brown liquor by, for example, alcohol precipitation, dialysis, ion exclusion, or ultrafiltration. The resulting sulfonated lignin is water‐soluble due to sulfonation of the reactive α‐positions, and the sulfonate groups increase the solubility, even at low pH. Similar to Kraft lignin, lignosulfonates are usually heavily degraded (i. e., condensation has taken place), which increases the C−C linkages and decreases the ratio of β‐O‐4 linkages. However, lignosulfonates have an even higher content of sulfur than Kraft lignin (3–8 wt %).^[38]^ Unlike the Kraft and the soda processes, sulfite pulping is performed in an acidic environment.

#### 2.3.4 Organosolv lignin

In organosolv pulping, lignin is extracted using an organic solvent, such as acetone, methanol, ethanol, or butanol, in the presence of water and an acid catalyst. Various organosolv methods have been reported in the literature, and the four main processes are the Organocell, Alcell, alkaline sulfite‐anthraquinone‐methanol, and acetosolv processes. The degree of structural modification is highly dependent on the severity of the process conditions; however, solubilization in the organic medium enables a lignin fraction with a low degree of structural alterations and dispersity to be obtained. Technical organosolv lignins obtained from the main industrial processes such as the Alcell process also lose the majority of their β‐O‐4 linkages.^[4]^

#### 2.3.5 Steam‐explosion lignin

Another acidic extraction method is steam explosion pretreatment, where the biomass is treated with pressurized steam/water. This is followed by an explosive release of the pressure, which causes disintegration of the lignocellulosic matrix. Lignin and hemicellulose can then be extracted with an organic or alkaline solution, followed by the precipitation of lignin, for example, by acidification. This method entails moderate to severe condensation, where 50–100 % of the β‐O‐4 linkages are lost.^[4]^

**Figure 5:**
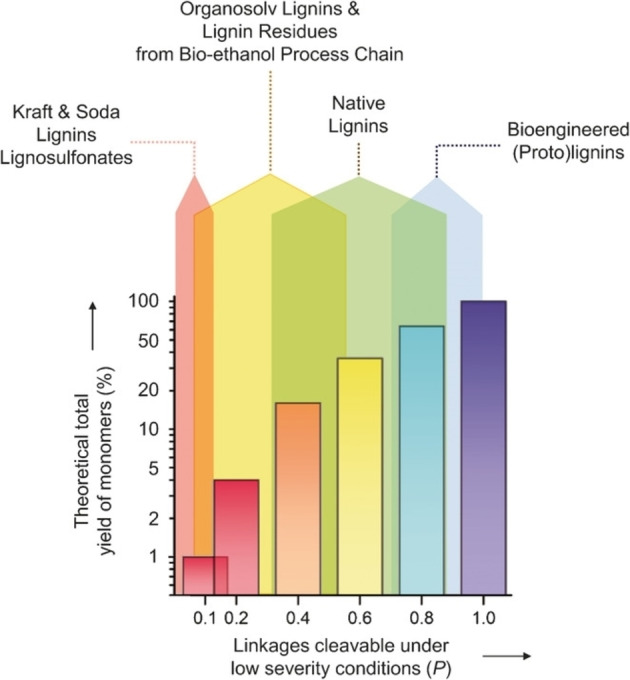
Theoretical monomer yield as a function of cleavable linkages in different lignins. Reproduced from Ref. [33], under the terms of the Creative Commons Attribution‐NonCommercial 4.0 International License (https://creativecommons.org/licenses/by‐nc/4.0/).

The theoretical monomer yield from different industrial, native, and bioengineered (proto)‐lignins as a function of the ability to cleave the bonds is illustrated in Figure 5. It is evident that the valorization of industrial lignins is considerably more challenging than the valorization of native lignins. However, the composition of lignins extracted using the same general method can vary substantially. A study by Barta and co‐workers showed that the overall β‐aryl ether content was very different in self‐produced organosolv lignins from that in technical lignins, reporting values in the range of 13–62 linkages per 100 C_9_ units for the former and of 2–13 for the latter.^[39]^ Such a difference is expected to significantly affect the reactivity to depolymerization of the different types of lignin due to the greater robustness of the C−C bonds than the ether bonds.

## 3 Oxidative Valorization of Lignin

### 3.1 Products from lignin

Due to its high content of aromatics, the lignin structure is also a prospective natural source of a range of aromatic chemicals, such as phenol, guaiacol (1‐hydroxy‐2‐methoxybenzene), and vanillin (4‐hydroxy‐3‐methoxybenzaldehyde). Other products that can be obtained from lignin include carbon fibers, activated carbon, and bioplastics. Lignin is also used directly as a binder in pellets or compressed materials, as a dispersant for soils, and in detergents, cement mixes, and other products.^[40]^ Phenol is an important precursor in a range of pharmaceuticals, herbicides, plastics, epoxy‐ and polyurethane resins, and various cosmetics. More than 95 % of the current phenol production is based on petroleum‐derived benzene. Guaiacol can be derived from guaiacum or wood creosote and is also produced from petrochemical‐based sources. It is used as a precursor for flavoring agents, including vanillin and eugenol (4‐allyl‐2‐methoxy‐phenol). Derivatives can be used as an antiseptic and local anesthetics.^[40]^

The oxidative depolymerization of lignin has historically focused on the production of vanillin, which is used as a food‐flavoring agent, in pharmaceuticals, and in the fragrance industry. Vanillin can be extracted from vanilla beans, but the demand vastly exceeds the vanilla bean supply, and synthetic methods have consequently been developed. The production of vanillin by the oxidative depolymerization of lignin, derived from the pulp and paper industry, was initiated in 1937 by the Salvo Chemical Corporation.^[41]^ Production was based on lignin extracted by sulfite pulping, which was the leading pulping technology at the time. This production then ceased due to the emergence of the Kraft pulping process, which led to a decrease in the number of sulfite pulping facilities, and vanillin was increasingly produced from the cheap petrochemical guaiacol, which covers about 85 % of the world's vanillin supply today.^[42]^ However, the need for alternatives to non‐renewable raw materials has encouraged research on the use of renewable feedstocks for vanillin production. At present, only a small amount of vanillin is produced from lignin derived from wood pulping. The Norwegian company Borregaard has been producing vanillin from lignin extracted by the lignosulfonate pulping process for more than 50 years and is currently the only company producing vanillin with this method, together with other lignin‐based performance chemicals.[^41^, ^43^]

### 3.2 Methods for depolymerization

Cleavage of the C−O and C−C bonds in the polymeric structure is necessary for the valorization of lignin. Catalysts are commonly employed for such processes to reduce the energy consumption, reaction time, temperature, and pressure, and to simultaneously obtain higher product yields and selectivity. A catalytic method frequently described in the literature is alkaline oxidative depolymerization. For example, Paananen et al. performed alkaline oxidative depolymerization of Kraft lignin from softwood to obtain oxidized lignin materials that can be used as a substitute for phenol in phenol‐formaldehyde resins.^[44]^ In their study, alkali‐catalyzed oxidation was selected due to the possibility of tuning the reaction conditions, for example, by varying the base concentration, temperature, and oxygen pressure, to obtain reactive, medium‐sized lignin‐derived molecules.

Several homogeneous catalysts capable of selectively oxidizing lignin have also been described in the literature, and many examples have been reviewed by Weckhuysen and co‐workers.^[20]^ The activity and stability of homogeneous catalysts can, to some extent, be customized to oxidize specific linkages in lignin due to the variety of ligands that can be implemented, including oxovanadium complexes, salen complexes, and metalloporphyrins. However, reported homogeneous catalysts also include metal ion systems without auxiliary ligands. For example, Wu et al. used a mixture of CuSO_4_ and FeCl_3_ to catalyze the alkaline oxidative depolymerization of steam‐explosion hardwood lignin, and the main monomeric products obtained were vanillin and syringaldehyde (4‐hydroxy‐3,5‐dimethoxy‐benzaldehyde), reaching maximum yields of 4.7 and 9.5 wt %, respectively.^[45]^ Bjørsvik and Minisci applied cobalt and copper complexes for the oxidative depolymerization of lignosulfonates to produce vanillin, where both metals were found to be effective in the oxidation by molecular oxygen.^[46]^ Ma et al. used CuSO_4_ as a catalyst for oxidative Kraft lignin depolymerization, and the synthesis of lignin‐phenol‐formaldehyde resin as a phenol substitute was again investigated as a potential application.^[47]^

Nonetheless, the greatest challenges when using homogeneous catalysts are their separation, regeneration, and recyclability. More cost‐ and separation‐efficient approaches are therefore required, and one possibility is the development of inexpensive heterogeneous catalysts.^[22]^ However, heterogeneous catalysts also pose challenges that must be overcome, for example, leaching, reduction in surface area, poisoning of the active sites, and coking of the catalyst surface,^[48]^ as described below.

Because of the structural complexity of polymeric lignin, many investigations have focused on model compounds, synthesized to mimic the ether and C−C bonds in real lignin. The model compounds employed are typically monomeric alcohols, such as veratryl alcohol (3,4‐dimethoxybenzyl alcohol) and vanillyl alcohol (4‐hydroxy‐3‐methoxybenzyl alcohol). Reactions employing model substrates can provide some insight into the cleavage of the various types of bonds present in lignin, as well as mechanistic insight. Model compounds can also be useful for screening reaction conditions. However, most of these studies generally report poor activity or monomer yields when the methods are translated to real lignin, since model compounds are not completely representative. Hence, a reaction that works with a model compound cannot be expected to work with real lignin, while in nearly all cases a reaction performing poorly with a convincing model compound will also fail on a real lignin substrate.[^14^, ^33^]

Several examples of the use of heterogeneous catalysts for the oxidative depolymerization of lignin model compounds can be found in the literature. Mate et al. oxidized a wide range of lignin model compounds, including vanillyl alcohol, veratryl alcohol, and *p*‐sinapyl alcohol with a heterogeneous Co_3_O_4_ catalyst.^[49]^ Strassberger et al. used Cu/Al_2_O_3_ and Cu/MgO–Al_2_O_3_ for the conversion of a range of dimers mimicking the β‐O‐4 linkages.^[50]^ Yadav and Garg carried out catalytic wet oxidation of ferulic acid [3‐(4‐hydroxy‐3‐methoxyphenyl)‐2‐propenoic acid] using a range of supported copper catalysts.^[51]^ Shilpy et al. reported vanillin as the major component (99.8 %) resulting from the oxidation of the model compound vanillyl alcohol under basic conditions using a heterogeneous CoTiO_3_ catalyst and H_2_O_2_ as the oxidant.^[52]^

Most of the studies carried out within this field have been performed using simple model compounds due to the high degree of complexity of technical lignin, such as the high C−C bond and sulfur contents, which could deactivate the catalyst and complicate conversion. Research works have also commenced to use more native lignins for similar reasons by directly converting biomass into lignin‐based aromatics and carbohydrates.[^15^, ^53^, ^54^] However, if the challenges associated with the conversion of technical lignin can be overcome, it would be possible to utilize the plentiful resource stream from the pulp and paper and biofuel industries, making the production of chemicals from lignin more economically attractive.

### 3.3 Oxidants for depolymerization

During oxidative depolymerization, lignin is converted in the presence of an oxidizing agent, which is typically O_2_ or H_2_O_2_. Oxidation can induce the cleavage of side chains, producing phenolic aldehydes and acids, but it can also cleave the aromatic rings in lignin, resulting in aliphatic carboxylic acids. A challenge in oxidative depolymerization is to achieve partial oxidation of the bonds while preserving the aromaticity in order to obtain higher‐value products.

The oxidant used for lignin conversion has a considerable impact on the product selectivity, as well as the yield of monomers, and oxidants of various strengths have been investigated for lignin degradation over the years. H_2_O_2_ is an efficient and inexpensive oxidant, but its highly oxidizing nature can cause ring‐opening of aromatics. Conversely, mild oxidants such as air, oxygen, and nitrobenzene partially oxidize the lignin substrate and do not disrupt the aromatic nature. Nitrobenzene has proven to be a highly selective oxidant for the conversion of lignin to aldehydes, but it cannot be used commercially due to its carcinogenic nature. Nevertheless, nitrobenzene has been used as the oxidant in several studies. For example, Wang et al. performed nitrobenzene oxidative depolymerization of Kraft, alkali, sodium lignosulfonate, swollen residual enzyme, and double enzymatic lignins, giving vanillin yields of 2.51, 6.52, 11.64, 13.39, and 9.17 %, respectively.^[55]^

Atmospheric air, with an O_2_ content of approximately 21 %, is a cheap, environmentally benign, and sustainable oxidant, but rather harsh conditions may be required to provide good results. Due to the low concentration of O_2_ in air, the pressure must be increased five‐fold to achieve the same concentration of dissolved O_2_ in solution. Pure O_2_ thus gives better results than air under milder conditions, while still being low in cost. It is readily available, and the main side‐product is water, making it a green and sustainable option. However, the use of pure oxygen is associated with some disadvantages; the first being the increased hazard, compared to air, due to its higher reactivity, and the second the rather weakly oxidizing nature of oxygen in its normal state. Highly alkaline conditions are therefore required when using oxygen as the oxidant to ionize the free phenolic hydroxyl groups. Last, but not least, the formation of radicals during the reaction is of concern and can cause repolymerization of fragments cleaved from the lignin structure or undesired reaction products.^[56]^

### 3.4 Heterogeneous catalysis for the oxidative valorization of technical lignin

A chronological overview of the studies reported in the literature on the oxidative depolymerization of various technical lignins into valuable products, primarily vanillin and other phenolic aldehydes, using heterogeneous catalysis is compiled in Table 3. This table also includes examples where the lignin source is “self‐produced” using conventional methods for lignin extraction, such as Kraft or organosolv. It should be borne in mind when comparing these results that self‐produced lignin is typically less condensed than lignin from industrial sources due to the difference in severity of the applied conditions.[^4^, ^39^]

**Table 3:** Overview of literature on the oxidative depolymerization of technical lignin using heterogeneous catalysts.

Entry	Catalyst	Solvent	Oxidant	Reaction conditions	Lignin type	Conv. [wt %]	Products	Yield [wt %]	Ref.
1	Pd/γ‐Al_2_O_3_	NaOH/H_2_O	O_2_	100–140 °C 2–10 bar 60 g lignin L^−1^ 2.5–5 wt % catalyst	organosolv (extracted from sugarcane bagasse)	–	vanillin syringaldehyde 4‐HBAL	≈12 (combined)	[57]
2	Pd/γ‐Al_2_O_3_	NaOH/H_2_O	O_2_	100–140 °C 2–10 bar 0–2 h 30–60 g lignin L^−1^ 2.5–5 wt % catalyst batch and flow	alkaline (extracted from sugarcane bagasse)	50–80	vanillin syringaldehyde	1.87–2.17^[a]^ 1.67–3.82^[a]^	[58]
3	LaMnO_3_	NaOH/H_2_O	O_2_	120 °C 5 bar O_2_+15 bar N_2_ 0–3 h 60 g lignin L^−1^	enzymatic hydrolysis of steam‐exploded cornstalk	41.8–57.0	vanillin syringaldehyde 4‐HBAL	4.32 9.33 2.03	[59]
4	LaFe_1‐*x*_Cu_*x*_O_3_ (*x*=0, 0.1, 0.2)	NaOH/H_2_O	O_2_	120 °C 5 bar O_2_+15 bar N_2_ 0–3 h 60 g lignin L^−1^	enzymatic hydrolysis of steam‐exploded cornstalk	41.8–66.6	vanillin syringaldehyde 4‐HBAL	4.56 11.51 2.49	[60]
5	LaCoO_3_	NaOH/H_2_O	O_2_	120 °C 5 bar O_2_+15 bar N_2_ 0–3 h 60 g lignin L^−1^ 5 wt % catalyst	steam‐exploded cornstalk	≈41–61	vanillin syringaldehyde 4‐HBAL	4.55 9.99 2.23	[61]
6	LaCo_1‐*x*_Cu_*x*_O_3_ (*x*=0, 0.1, 0.2)	NaOH/H_2_O	O_2_	120 °C 5 bar O_2_+15 bar N_2_ 0–3 h 60 g lignin L^−1^ 5 wt % catalyst	steam‐exploded cornstalk	≈55–65	vanillin syringaldehyde 4‐HBAL	4.76–5.30 11.0–12.80 2.71–2.88	[62]
7	CuFeS_2_	CH_3_COOH/CH_3_COONa/H_2_O	H_2_O_2_	60 °C 0–5 h 50–100 g lignin L^−1^ 10 wt % catalyst	biorefinery (DACSL and SESPL)	–	dicarboxylic acids^[c]^	14^[b]^ 11^[c]^	[63]
8	Co[H_*x*_]salen/NaY (*x*=2 or 4)	H_2_O or H_2_O/CH_3_OH	peracetic acid	70 °C 3 h 10 g lignin L^−1^ 20 wt % catalyst	Kraft	6.5–23.3^[d,e]^ 6.9–46.0^[d,f]^	2‐methoxyphenol 2‐hydroxybenzaldehyde 4‐hydroxy‐3,5‐dimethoxyphenyl ethanone	–	[64]
9	Cu−V‐hydrotalcites (HTc)s	pyridine	O_2_	135 °C 10 bar O_2_ 40 h 22.22 g lignin L^−1^ 20 wt % catalyst	organosolv Kraft	–	–	–	[65]
10	Cu[H_*x*_]salen/NaY (*x*=2 or 4)	H_2_O or H_2_O/CH_3_OH	peracetic acid	70 °C 3 h 10 g lignin L^−1^ 20 wt % catalyst	Kraft	–	2‐methoxyphenol 4‐HBAL 4‐hydroxy‐3, 5‐dimethoxyphenyl ethanone	–	[66]
11	Pd/CeO_2_	CH_3_OH	O_2_	185 °C 1 bar O_2_ 24 h 2 g lignin L^−1^ 200 wt % catalyst	organosolv	–	vanillin guaiacol 4‐HBAL	5.20 0.87 2.40	[67]
12	Co(salen)/graphene oxide (GO)	acetonitrile/THF	air	80 °C 24 h 1 g lignin L^−1^ 10 wt % catalyst	organosolv	–	vanillin vanillic acid 1‐(4‐hydroxy‐3‐methoxyphenyl) ethanol	3.07	[68]
13	CuCl_2_/polybenzoxazine (PBOZ)	CH_3_CN and NaOH	H_2_O_2_	RT 8–24 h 25 g lignin L^−1^ 18 wt % catalyst	dealkaline	–	–	–	[69]
14	HTc−Cu‐V	pyridine	O_2_	135 °C 5 bar 20 wt % catalyst	Kraft	–	vanillin vanillic acid	–	[70]
15	CoFeO	H_2_O and H_2_O/CH_3_OH	O_2_	150–200 °C 1–2 bar (10 % O_2_ in N_2_) 2–4 h 1.66 g lignin L^−1^ 20–40 wt % catalyst	organosolv	40.2^[a]^ 55.6^[b]^	aromatic molecules	19.6^[g]^ 16.6^[h]^	[71]
16	Au/CeO_2_	CH_3_OH	O_2_	180 °C 10 bar 4 g lignin L^−1^ 20 wt % catalyst	organosolv	–	vanillin methyl vanillate methylene syringate	10.5 6.8 3.4	[72]
17	Cu−Mn/ δ‐Al_2_O_3_	H_2_O	O_2_	160 °C 15 bar 20 g lignin L^−1^ 50 wt % catalyst	lignosulfonate	–	vanillin 4‐HBAL vanillic acid 4‐HBA	–	[73]
18	Co/ZrO_2_ Co/TiO_2_ Co/CeO_2_	CH_3_OH	O_2_	140 °C 5 bar 1 h 79.2 g lignin L^−1^ 10 wt % catalyst	alkali	54.4^[i]^ 61.4^[j]^ 64.6^[k]^	vanillin 4‐hydroxy‐4‐methyl‐2‐pentanone	–	[74]
19	Cu−Mn mixed oxides	NaOH/H_2_O	H_2_O_2_	120–180 °C atmospheric pressure 1 h 10 g lignin L^−1^ 10 wt % catalyst	Kraft	–	vanillin	6.8^[d]^	[75]
20	MoPO/CeO_2_	NaOH/H_2_O	O_2_	150 °C 5 bar 3 h 33.33 g lignin L^−1^ 20 wt % catalyst	alkali	68	vanillin acetovanillone vanillic acid guaiacol	9.0 1.6 1.3 0.6	[76]
21	Ce−Cu/MFI nanosheets	CH_3_CH_2_OH	O_2_	150 °C 10 bar 24 h 20 g lignin L^−1^ 100 wt % catalyst	organosolv	81.6^[a]^	diethyl maleate C_3_−C_5_ esters aromatics	18.1 6.2 5.1	[77]
22	Au/TiO_2_ and Pt/TiO_2_	NaOH/H_2_O	air	150 °C 20 bar 0.5–1 h 5 g lignin L^−1^ 0.1 wt % metal	organosolv (hardwood) Kraft organosolv (softwood)	–	vanillin	1.7^[l]^ 3.4^[m]^ 5.1^[n]^	[78]
23	Pd‐PVP/TiO_2_ and Pd/TiO_2_	NaOH/H_2_O	air	150 °C 20 bar 5 g lignin L^−1^ 1–10 wt % metal	Kraft	50–73	vanillin vanillic acid	1.5–2.5 1–2	[79]

[a] Yields calculated from concentrations. [b] DACSL=diluted‐acid corn stover lignin. [c] SESPL=steam‐exploded spruce lignin. [d] Not indicated if it is in wt %. [e] Co[H_2_]salen. [f] Co[H_4_]salen. [g] H_2_O, 150 °C. [h] H_2_O+CH_3_OH, 200 °C. [i] Co/ZrO_2_; [j] Co/TiO_2_. [k]Co/CeO_2_. [l] Hardwood organosolv. [m] Kraft. [n] Softwood organosolv.

#### 3.4.1 Supported noble‐metal catalysts

Sales et al.[^57^, ^58^] conducted studies on the conversion of lignin obtained from sugar cane bagasse by the organosolv acid process and alkaline extraction (Table 3, entries 1 and 2). Oxidative depolymerization was assisted by an alumina‐supported palladium catalyst giving various aldehyde products such as vanillin, syringaldehyde, and *p*‐hydroxybenzaldehyde (4‐HBAL). Alkaline lignin conversion was investigated in both batch and continuous processes, and it was found that higher yields were obtained from the continuous process. The maximum yield of vanillin obtained from the continuous reaction was 6.51 g, from a solution containing 30 g lignin L^−1^ at a flow rate of 5 L h^−1^ for 2 h, corresponding to 2.17 wt % with respect to the lignin feed. This is relatively low compared to later reports, as can be seen in Table 3. The recyclability of this catalytic system was not reported.

Deng et al.^[67]^ screened Pd catalysts supported on Al_2_O_3_, SiO_2_, MgO, and CeO_2_ for the oxidation of the lignin model compound 2‐phenoxy‐1‐phenylethanol (PP‐ol) using molecular O_2_ as the oxidant. The CeO_2_‐supported Pd nanoparticles were the most promising system, and this system was used for the conversion of organosolv lignin to vanillin, guaiacol, and 4‐HBAL (Table 3, entry 11). The source of the organosolv lignin was, however, not given. A relatively high vanillin yield of 5.2 wt % was achieved, with a catalyst/substrate ratio of 2 : 1, so the result cannot justifiably be compared to other results given in Table 3. The stability of the Pd/CeO_2_ catalyst was tested in the oxidation of PP‐ol, showing no significant differences in conversion. However, a shift in the reaction products was observed. No information was given on the stability of the catalyst after oxidative depolymerization of the organosolv substrate.

Au/CeO_2_ was used as a catalyst by Song et al.^[72]^ for the oxidative depolymerization of self‐extracted organosolv lignin in methanol using molecular O_2_ as the oxidant (Table 3, entry 16). Organosolv lignin was prepared through an ethanol‐based organosolv process. The reactions were carried out without the use of a strong base. The product mixture was analyzed with gas chromatography–mass spectrometry (GC–MS) and revealed a range of aromatic monomers, with an impressive yield of 10.5 wt % vanillin, 6.8 wt % methyl vanillate, and 3.4 wt % methyl syringate, together with a ring‐opened species. The stability of the Au/CeO_2_ catalyst was tested in the oxidation of PP‐ol. After the catalyst had been used four times, only a minor reduction in conversion was observed. However, a slightly greater decrease in lignin conversion was observed in the fifth and the sixth runs. No information was provided on the recyclability of the catalyst used for the oxidative depolymerization of organosolv lignin.

In a recent study by Cabral Almada et al.,^[78]^ Au and Pt particles supported on TiO_2_ were screened as catalysts for oxidative depolymerization of softwood Kraft lignin and hard‐ and softwood organosolv lignins in NaOH solution using air as the oxidant (Table 3, entry 22). The organosolv lignins were self‐extracted with the ethanol organosolv process, and the Kraft lignin was obtained from black liquor from the pulp and paper industry. The Au/TiO_2_ catalyst yielded ring‐opened products due to over‐oxidation and was deemed unsuitable for the production of aromatic products. The Pt/TiO_2_ catalyst gave higher yields of aromatic products, mainly vanillin and syringaldehyde. The catalyzed depolymerization reactions of Kraft, softwood organosolv and hardwood organosolv lignins afforded 3.4, 5.1, and 1.7 wt % vanillin, respectively. The reaction media were analyzed after the oxidative depolymerization reaction catalyzed by Pt/TiO_2_ revealing that 18 wt % of the metal had leached into the solution, implying that the catalyst was not stable under the reaction conditions used.

In a follow‐up study by Bourbiaux et al.,^[79]^ Pd‐based catalysts were prepared and tested in the oxidative depolymerization of Kraft lignin into aromatic monomers (Table 3, entry 23). Kraft lignin was isolated from a *Pinus pinaster* black liquor from the pulp and paper industry, and the reactions were carried out using aqueous NaOH as the solvent under an air atmosphere. Relatively high lignin conversion was achieved (50–73 wt %) with catalysts involving Pd nanoparticles stabilized in water by polyvinylpyrrolidone (PVP) or supported on TiO_2_. The Pd/TiO_2_ catalyst resulted in yields of up to approximately 2.5 wt % vanillin, 1.5 wt % vanillic acid (4‐hydroxy‐3‐methoxybenzoic acid), and 0.7 wt % acetovanillone (4‐hydroxy‐3‐methoxyacetophenone), with the selectivity for vanillin reaching 55 %. However, different results were obtained using the Pd‐PVP/TiO_2_ catalyst, where the highest selectivity observed was for vanillic acid (45 %), and this was attributed to a difference in the palladium accessibility and/or stability. No data were reported on the recycling or reuse of the catalysts investigated, which is especially important when dealing with noble‐metal‐based catalysts.

#### 3.4.2 Metal oxide catalysts

Perovskite‐type mixed oxide catalysts have been used by Deng et al. and Zhang et al.[^59^, ^60^, ^61^, ^62^] for the conversion of self‐produced steam‐exploded lignin by catalytic wet air oxidation (Table 3, entries 3–6). In the first three studies, lanthanum‐based perovskites LaMnO_3_, LaCoO_3_, and LaFe_1‐*x*_Cu_*x*_O_3_ (*x*=0, 0.1, 0.2) prepared by the sol–gel method were employed as catalysts. These three catalysts showed high activity in the oxidative conversion of lignin, compared to the non‐catalytic process. The maximum conversion of lignin in the catalytic reactions was in the range of 57.0–66.6 wt % and gave vanillin yields in the range 4.32–4.56 wt %. Furthermore, these perovskite catalysts showed sufficiently high stability to allow recycling without any significant change in lignin conversion.[^59^, ^60^, ^61^] In a fourth study using the perovskite‐type mixed oxide LaCo_1‐*x*_Cu_*x*_O_3_ (*x*=0, 0.1, 0.2), Deng et al. concluded that the yields of aromatic aldehydes increased when the Co‐based structure was doped with Cu, and that the effect became more pronounced when increasing the amount of Cu.^[62]^ The highest vanillin yield, of 5.3 wt %, was obtained when employing LaCo_0.8_Cu_0.2_O_3_ as the catalyst. Based on this observation, a mechanism was proposed suggesting that an anion vacancy was formed by Cu incorporation, allowing more oxygen to adsorb onto the surface. No data were reported on the recyclability of the LaCo_1‐*x*_Cu_*x*_O_3_ catalyst.

Hdidou et al.^[71]^ also applied various metal oxides for oxidative lignin depolymerization (Table 3, entry 15). Different Co–Fe oxide catalysts were screened using lignin model compounds, and the highest conversion was obtained with a CoFeO (Co/Fe=1) catalyst, subsequently applied in oxidative depolymerization of organosolv lignin with O_2_ as oxidant. In the majority of these studies, a strongly alkaline aqueous solution or a purely organic solvent was employed. However, both water and a mixture of methanol and water were assessed as the solvent. The highest conversion of lignin was obtained for the water–methanol mixture with 20 wt % catalyst at 200 °C for 4 h. The residual lignin content was at 44.4 wt % and the aromatic molecule yield was 16.6 wt %. The selectivity was highest to syringaldehyde (38.84 %), followed by vanillin (12.61 %). The highest yield of aromatic molecules (19.6 wt %) was obtained with 20 wt % catalyst in water at 150 °C for 4 h. This reaction also exhibited the highest selectivity to syringaldehyde (49.77 %) and vanillin (17.06 %). However, the reaction resulted in a lower lignin conversion than in the water–methanol reaction, as the residual lignin was 59.8 wt %. The CoFeO (Co/Fe=1) catalyst was reused twice to test its stability, and no significant changes in the aromatic yield or selectivity were observed. The conversion during the recycling runs was not given.

**Figure 6:**
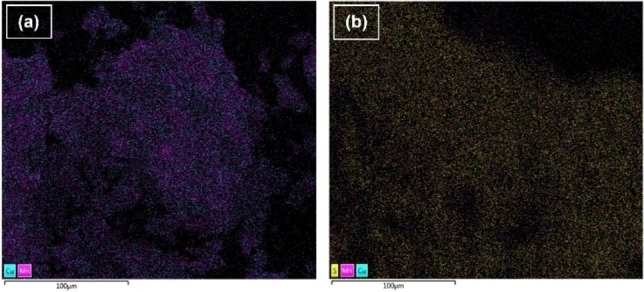
Elemental mapping of (a) fresh and (b) spent Cu−Mn catalyst by scanning electron microscopy–energy dispersive X‐ray spectroscopy (SEM–EDX). A significant increase can be seen in the amount of sulfur (yellow) in the spent catalyst, compared to the fresh catalyst. The signal intensities of Cu (aqua) and Mn (pink) also appeared to be lower in the spent catalyst. Scale bars: 100 μm. Adapted from Ref. [73], under the terms of the Creative Commons Attribution 4.0 International License (http://creativecommons.org/licenses/by/4.0/).

Cu−Mn and Ni−Mo oxides supported on δ‐Al_2_O_3_ were used by Abdelaziz et al.^[73]^ in the oxidative depolymerization of sodium lignosulfonates (Table 3, entry 17). Since lignosulfonates are water‐soluble, the reactions were carried out in an aqueous solution, and molecular O_2_ was employed as the oxidant. Size‐exclusion chromatography was used to determine the molecular weight distributions of the different lignin fractions. It was observed that the Cu−Mn‐based catalyst was superior to a Ni−Mo system in converting the substrate into low‐molecular‐weight aromatic products. The higher catalytic activity of the Cu−Mn catalyst was explained by the ability of Mn and Cu to form highly‐oxidized oxo–metal complexes when reacted with oxygen. The catalytically depolymerized sample contained vanillin, 4‐HBAL, vanillic acid, and 4‐hydroxybenzoic acid (4‐HBA). However, specific yields and lignin conversion were not given. Characterization of the fresh and the spent catalysts revealed significant leaching of Cu and Mn, and increased sulfur content in the spent catalyst (Figure 6). The instability of the catalyst system reduced its recyclability.

Kumar et al.^[74]^ used Co supported on TiO_2_, CeO_2_, and ZrO_2_ to study the effect of the catalyst support on the oxidative depolymerization of alkali lignin with O_2_ as oxidant (Table 3, entry 18). The highest bio‐oil yield from the oxidative depolymerization of alkali lignin (60.2 wt %) and the highest lignin conversion (64.6 wt %) were obtained with the Co/CeO_2_ catalyst, which could be explained by a larger pore diameter, as well as better cobalt distribution on the CeO_2_ support than on the TiO_2_ and ZrO_2_ supports. Vanillin was the major monomeric product found in the bio‐oil, followed by 4‐hydroxy‐4‐methyl‐2‐pentanone. No information was reported on the stability or recyclability of any of the three catalytic systems.

**Figure 7:**
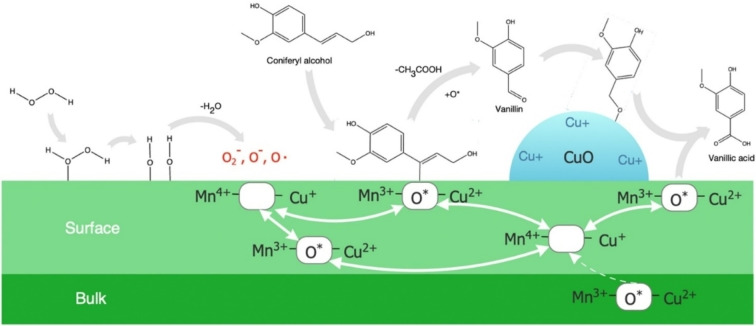
Proposed reaction pathway for oxidative lignin conversion into vanillin and vanillic acid over Cu‐based catalysts. Assuming that the starting compound for the production of vanillin from lignin is coniferyl alcohol, the oxidizing agent H_2_O_2_ is dissociatively adsorbed onto active Cu−Mn sites on the catalyst surface and decomposed to O_2_, which is then transformed into surface oxygen species (O*) at the oxygen vacancy (Mn^4+^−Ο*−Cu^+^), leading to the formation of Mn^3+^−O*−Cu^2+^. The lignin‐derived coniferyl alcohol is converted to vanillin by means of the surface oxygen species, where the vanillin produced can be oxidized to vanillic acid, depending on the Cu content in the catalyst. Reproduced with permission from Ref. [75]; Copyright 2020, Elsevier.

Likewise, Jeon et al.^[75]^ tested mixed oxides of Cu and Mn for the conversion of commercially purchased Kraft lignin through alkaline oxidative depolymerization, with H_2_O_2_ as the oxidant, at temperatures of 120–180 °C (Table 3, entry 19). Five different Cu/Mn ratios were investigated, as well as pure Cu and Mn oxides. X‐ray diffraction (XRD) was used to determine the crystal structures of the prepared metal oxide catalysts, showing that the pure Mn oxide had two crystal forms: MnO_2_ and Mn_2_O_3_, while the mixed oxides had a non‐stoichiometric cubic spinel structure Cu_*x*_Mn_3‐*x*_O_4_. With a ratio of Cu/Mn of 1 : 3, the only structure observed was Cu_1.5_Mn_1.5_O_4_, whereas a CuO phase was observed along the spinel structure with Cu/Mn ratios of 1 : 1 and 3 : 1. Analysis with high‐performance liquid chromatography (HPLC) revealed vanillin, syringaldehyde, vanillic acid, syringic acid, acetovanillone, and 4‐HBAL as the main aromatic products. The CuMn(1 : 3) mixed oxide proved to have the best catalytic effect, but the highest yield of monomeric products was obtained at 180 °C in all the systems investigated. Notably, the highest yield of vanillin (6.8 %) was obtained at a temperature of 150 °C, suggesting that vanillin was unstable at higher temperatures, and thus over‐oxidized into organic acids. The good catalytic performance of the CuMn(1 : 3) oxide catalyst was attributed to improved redox properties compared to the other oxides. This interpretation was supported by oxygen‐temperature‐programmed desorption, which indicated higher oxygen mobility of this catalyst. Based on information related to catalyst structure and product behavior under the reaction conditions tested, a reaction pathway for the oxidative lignin conversion into vanillin and vanillic acid over Cu‐based catalysts was proposed (Figure 7). No information was provided on catalyst stability and recyclability.

Rawat et al.^[76]^ recently reported on the use of molybdenum pyrophosphate supported on CeO_2_ (MoPO/CeO_2_) for the oxidative depolymerization of commercial alkali lignin for the selective production of vanillin (Table 3, entry 20). The reaction catalyzed by MoPO/CeO_2_ yielded 12.5 wt % total monomers, including 9 wt % vanillin, 1.6 wt % acetovanillone, 1.3 wt % vanillic acid, and 0.6 wt % guaiacol, using O_2_ as the oxidant in an aqueous NaOH solution, where an optimum concentration of 2 m NaOH was established together with optimum reaction conditions of 150 °C and 3 h. This result is very promising compared to many other results in the literature, but the recyclability of the catalyst proved poor as the vanillin yields decreased to 3.5 wt % in the third reaction cycle. N_2_ physisorption measurements on the fresh and spent catalysts showed a severe reduction in surface area of the MoPO/CeO_2_ catalyst after reaction (from 150 to 10 m^2^ g^−1^). Thermogravimetric analysis indicated the presence of organic material on the spent catalyst. Both these findings suggested clogging of the catalyst pores with organic material derived from lignin, explaining the decrease in catalytic activity. However, the recycling reactions were performed without intermediate catalyst calcination, which could probably have removed some of the deposits of organic material and improve recyclability.

Ce−Cu oxides supported on hierarchical MFI zeolite nanosheets were used by Li et al.^[77]^ for the selective oxidative depolymerization of organosolv lignin through oxidative cleavage (Table 3, entry 21). MFI zeolite nanosheets were used as they had proven to be suitable in catalytic reactions with bulky substrates, as is the case for lignin. CuO was chosen due to its documented catalytic activity, while CeO_2_ was used as a promoter for the catalyst due to its facile ability to shift between Ce^IV^ and Ce^III^ as well as the high number of oxygen vacancies, affording high oxygen mobility. Metal loading was screened (5–15 wt % Ce with 5 wt % Cu) as well as unloaded MFI nanosheets, pure metal oxides, mixed metal oxides, and metal oxides on other zeolite supports (ZSM‐5 and Al‐SBA‐15). The highest lignin conversion (81.6 wt %) was obtained with a loading of 15 wt % Ce and 5 wt % Cu on the MFI nanosheets, at 150 °C after 24 h, with a catalyst loading of 100 wt % (with respect to the lignin substrate). The reaction produced various esters (6.2 wt %), including ethyl levulinate, diethyl fumarate, diethyl succinate, and diethyl malate, as well as various aromatics (5.1 wt %), including benzaldehyde, ethyl benzoate, 1‐naphthalenol, benzoic acid, vanillin, ethyl vanillate, 4‐HBA, and ethyl mandelate. The recyclability of the catalyst was investigated, however, in the conversion of the β‐O‐4 lignin model 2‐phenoxy‐1‐phenylethanone. The catalytic activity declined considerably after the fourth run, but the catalyst performance was retained after recalcination. Carbon deposition and mechanical loss were thought to be the principal reasons for the decrease in catalytic activity.

#### 3.4.3 Supported complex catalysts

A different approach was applied by Zhou^[64]^ for selective, oxidative lignin depolymerization with heterogeneous catalysis using supported cobalt [H_2_]salen and [H_4_]salen complexes (Table 3, entry 8). The salen‐type complexes constitute a class of compounds of great importance due to their simple synthesis and high catalytic activity in a range of oxidation reactions with oxidants such as oxygen and hydrogen peroxide; properties making them potential biomimetic catalysts for oxidative lignin depolymerization. Catalytic oxidative depolymerization reactions were carried out on self‐produced Kraft lignin from *eucalyptus*, and to avoid catalyst deactivation, the Co[H_2_]salen and Co[H_4_]salen complexes were encapsulated in NaY zeolites, which endowed them with heterogeneous behavior. Different methods of encapsulation were investigated, and the Co[H_4_]salen‐encapsulated complex prepared by impregnation was found to be the most active for Kraft lignin conversion in water and methanol, using peracetic acid as the oxidant. Under such conditions, the catalyst yielded the highest lignin conversion (46 %) and high selectivity for 2‐methoxyphenol, while other major products included 2‐hydroxy benzaldehyde and 4‐hydroxy‐3,5‐dimethoxyphenyl ethanone.

In another study by Zhou,^[66]^ the corresponding copper [H_*x*_]salen complex, also encapsulated in NaY, was investigated for the selective oxidative depolymerization of self‐produced Kraft lignin (Table 3, entry 10). The Cu[H_4_]salen complex prepared by the “ship‐in‐a‐bottle” method had the highest lignin conversion (8274.63 g mol^−1^ of Cu), and it was reported that this catalytic system also had high selectivity for the formation of 2‐methoxyphenol. Other products included 4‐HBAL, and 4‐hydroxy‐3‐methoxy benzoic acid, as well as small amounts of vanillin, 4‐hydroxy‐3‐methoxy benzoic acid, and 2,6‐dimethoxybenzoquinone. Neither the robustness nor the recyclability of the catalysts was reported in the studies on the NaY‐supported Co‐ and Cu‐[H_4_]salen systems.

A third study by Zhou and Lu^[68]^ investigated Co(salen) supported on graphene oxide (GO) and other supports for the oxidative conversion of self‐produced organosolv lignin using air as the oxidant (Table 3, entry 12). GO was chosen as the support material due to its high specific surface area and chemical stability. The monomeric products were detected with GC–MS. Vanillin was the main product, and other products included vanillic acid and 1‐(4‐hydroxy‐3‐methoxyphenyl) ethanol. A corresponding homogeneous Co(salen) system was also investigated, but this system gave a lower yield than the heterogeneous catalyst. This was explained by the ability of the system to shift to more active species of the Co complex by uniformly dispersing it on the GO support. Other supports were also screened (NaY, MCM‐48, SSZ‐13), but the GO‐supported system showed better vanillin yields than the other supports. The maximum yield of vanillin obtained with the Co(salen)/GO catalyst was 3.07 wt %, with respect to the lignin feed. The recyclability of the catalysts was tested by recovering the spent catalyst from the reaction mixture and reusing it a further ten times. The Co(salen)/GO catalyst proved to be more stable than the other catalysts tested but showed a gradual decrease in performance over the ten consecutive runs, resulting in a final vanillin yield of approximately 2.8 wt % of the lignin feed. Acetonitrile was used as the solvent, which is harmful to the environment, and therefore cannot be regarded as an optimal choice.^[80]^

#### 3.4.4 Other catalytic systems

Much of the research concerning oxidative lignin depolymerization has focused on the production of low‐molecular‐weight aromatic compounds, such as vanillin, vanillic acid, and 4‐HBAL. In contrast, Ma et al.^[63]^ studied the selective production of dicarboxylic acids (DCAs) by the oxidative depolymerization of diluted‐acid corn stover lignin and steam‐exploded spruce lignin using chalcopyrite (CuFeS_2_) catalysts with H_2_O_2_ as the oxidant (Table 3, entry 7). The DCAs succinic and maleic acid were especially targeted in this study, as they are valuable industrial chemicals currently produced from petroleum‐based feedstock. The possibility of different oxidation states of the two transition metals in chalcopyrite (i. e., Cu^I^/Cu^II^ and Fe^II^/Fe^III^) enables effective electron transfer, and for this reason, it has potential as an effective catalyst in oxidation reactions in general. In line with this, it was observed that the catalytic system achieved ring opening of the aromatic rings at longer reaction times (>2 h), favoring the production of DCAs; the highest yield being obtained after 5 h. However, no information was provided on the stability or recyclability of the heterogeneous catalyst.

Mottweiler et al.^[65]^ synthesized heterogeneous catalysts based on transition‐metal‐containing hydrotalcites (Table 3, entry 9). Hydrotalcites (HTc) were chosen for their oxidation potential and were prepared by co‐precipitation. Three different solvents were screened (toluene, pyridine, and dimethyl carbonate), as was a range of transition metal HTcs (Co, Zn, Fe, Cu, and V) in the depolymerization of the lignin model compound dilignol with O_2_ as oxidant. High conversions (>99 %) of the model compound were observed for the HTc‐Cu−V and HTc‐Fe−V catalysts in pyridine. Once the reaction conditions had been optimized, the approach was applied to Kraft lignin. Products with molar masses below that of the initial lignin polymer were obtained, but the nature and yields of these products were not further specified. The best solvent was found to be pyridine, which is considered a problematic and possibly hazardous solvent that should be replaced to achieve a green reaction.^[81]^ The recyclability of the catalytic system was tested in the conversion of the model compound, showing that conversion was significantly decreased in the third run, likely due to leaching. A study using the same type of catalytic system for the oxidative depolymerization of commercial Kraft lignin was later reported by Rinesch and co‐workers (Table 3, entry 14). However, the main focus of their study was the cleavage of the β‐O‐4 linkage, and insight was gained mainly from the oxidative depolymerization of lignin model compounds.^[70]^

A new type of heterogeneous catalyst was devised by Ren et al.^[69]^ for mild oxidative lignin depolymerization with H_2_O_2_ at room temperature and ambient pressure (Table 3, entry 13). The catalyst was a composite based on Cu^II^ and the polymer polybenzoxazine (CuCl_2_/PBOZ). PBOZ was chosen due to its high thermal and chemical stability. Screening for optimum conditions in the conversion of chalcone lignin model compounds suggested that the catalyst was able to cleave both C−C and C−O bonds. This observation was confirmed using commercially purchased dealkaline lignin, where the degree of depolymerization was determined by gel permeation chromatography (GPC) after reaction with the catalytic system. The initial lignin sample had a molar mass of 2609 Da, whereas molar masses of 500 and 395 Da were found after 8 and 24 h of reaction, respectively. No conversion or monomer yields were reported for the oxidative depolymerization of dealkaline lignin, and catalyst recycling tests were only carried out with the model compound chalcone, showing that the selectivity to aromatics remained unchanged, but the conversion decreased significantly over three cycles.

In summary, oxidative depolymerization of lignin with heterogeneous catalysis has considerable potential for the selective production of value‐added compounds, including aromatic monomers, provided a number of challenges can be overcome. Several studies have reported the use of supported noble‐metal catalysts for the oxidative depolymerization of technical lignin. However, a serious drawback of noble metals is their cost, and this approach to the valorization of technical lignin streams may thus not be economically viable. Another challenge associated with heterogeneous catalysts is their recyclability, as it is important that the catalysts can be recovered and reused. However, few studies have reported good catalyst reusability, and information on catalyst stability is often not provided. In many cases, only the oxidation of model compounds has been studied, whereas it is necessary to test catalyst reusability in the oxidative depolymerization of real lignin streams.

A more general challenge arises in comparing the findings from different studies as disparate reaction parameters are often used. The comparison of studies involving radically different catalyst loadings, which can range from 2.5 to 200 wt % based on lignin input, is especially problematic. A further obstacle to the comparison of findings is the lack of a dedicated protocol for reporting the results. Some studies focus solely on the change in molecular weight distribution, some only report the selectivity to different products, while others report on conversion, bio‐oil yield, monomer yield, or yields of specific compounds such as vanillin. A more uniform protocol would thus be beneficial for comparing the findings of different studies.

### 3.5 Analytics in the oxidative valorization of technical lignin

Recent advances towards more sustainable chemistries rely on the availability of advanced analytical methods, making it easier and faster to visualize reaction outcomes and mechanistic details in the conversion of bio‐sourced material. The ability to avoid working blindly is thus central if new methods are to be developed rapidly that can compete with petroleum‐ and petrochemical‐based methods that have evolved over more than a century. Depending on the type of substance, the reaction, and the research question under scrutiny, various complementary physicochemical methods can provide detailed insight into the catalytic process and its outcome. These methods can be grouped into approaches that (pre)purify complex post‐reaction material through chromatographic analysis, and those (mostly spectroscopic) that detect chemical functionality in the unpurified post‐reaction material. The most suitable method will depend on the relative importance of reproducibility, accurate quantification, compound identification (including new chemicals), throughput, resolution, sensitivity, solvent and salt content, the distinction of compound classes with different volatility, rapid sample preparation, and so on.

#### 3.5.1 Chromatographic methods

The volatile monomers of interest in the oxidative depolymerization of technical lignins are often identified and quantified by GC analysis and comparison to authentic standard compounds. Detection is often achieved through MS or flame ionization detection (FID), or a combination of both.[^57^, ^58^, ^63^, ^64^, ^68^] Various responses, not least in FID, necessitate the determination of analyte‐specific response factors. Alternative approaches have included liquid chromatography (LC), which also involves calibration with authentic reference standards for identification and quantification.[^59^, ^60^, ^61^, ^62^, ^67^, ^72^]

HPLC approaches are common in the identification of aliphatic organic acids that are formed in alkaline aerobic lignin oxidation.[^13^, ^16^] LC has the benefit of being applicable to less volatile compounds, and places fewer demands on sample preparation than GC, but it is often considered to be the lower‐resolution and less reproducible chromatographic method. The versatility of chromatographic and detection methods is being increasingly employed for the determination of products and fragmentation pathways in technical lignins. For example, ultra‐ HPLC coupled with high‐resolution multiple‐stage tandem mass spectrometry (HRMS^n^) has been used to identify lignin‐oligomer fragmentation pathways in Kraft lignin.^[82]^ Supercritical fluid chromatography, often coupled with MS, is gaining in popularity due to the promise of resolving many compounds quickly with a putatively smaller matrix effect than LC–MS, while using a small amount of a benign solvent for separation.[^73^, ^83^]

Two‐dimensional GC methods using two columns of different polarity have been applied to improve the resolution and sensitivity compared to conventional GC–MS methods for diverse analyte classes in bio‐sourced oils.[^9^, ^84^, ^85^] The comprehensive identification of compounds in the gas phase has been achieved by μ‐GC. GPC is commonly employed for the analysis of lignin degradation and the identification of average molar masses. It has also recently been demonstrated that it is possible to obtain information on molecular weight in real time during technical lignin depolymerization by combining operando attenuated total reflectance infrared (ATR‐IR) spectroscopy with chemometrics.^[86]^ In the studies on oxidative depolymerization of technical lignins by heterogeneous catalysts, as summarized in Table 3, HPLC and GC separation coupled with FID, UV detection, refractive index, or MS detection were used in the majority of cases for the quantification of a few selected products that are commercially available as authentic standards.

#### 3.5.2 Spectroscopic methods

HRMS and high‐resolution spectroscopic frequency measurements offer a complementary approach to chromatography for tracking chemical changes in the degradation of lignin.^[87]^ ^1^H nuclear magnetic resonance (NMR) spectroscopy is mainly useful only for identification of the main reaction products and the emergence or loss of functional groups, as the post‐reaction mixture resulting from the conversion of technical lignin usually yields congested 1D ^1^H NMR spectra. Such analyses are occasionally complemented with the description of functional groups using Fourier‐transform IR spectroscopy.^[74]^ A successful approach for the characterization of functional groups in lignin‐sourced phenolics is the chemical instalment of NMR‐active spin‐1/2 nuclei with high natural abundance, such as ^31^P or ^19^F.[^88^, ^89^] Due to the low natural abundance of ^13^C, long measurement times are required using ^13^C NMR spectroscopy for the analysis of lignin and lignin‐sourced products through the accumulation of experimental repetitions. The sensitivity of these experiments increases with the square root of the number of repetitions, thus often leading to long reaction times for intrinsically insensitive analyses. 1D NMR experiments can be quantitative without uncertainty in the signal area if the repetitions are slow relative to the time constant (T_1_) for establishing equilibrium nuclear magnetization. Under these conditions, each NMR active nucleus of the same isotope yields the same response, irrespective of the analyte. However, the demand for slow repetitions implies slow measurements.^[90]^

**Figure 8:**
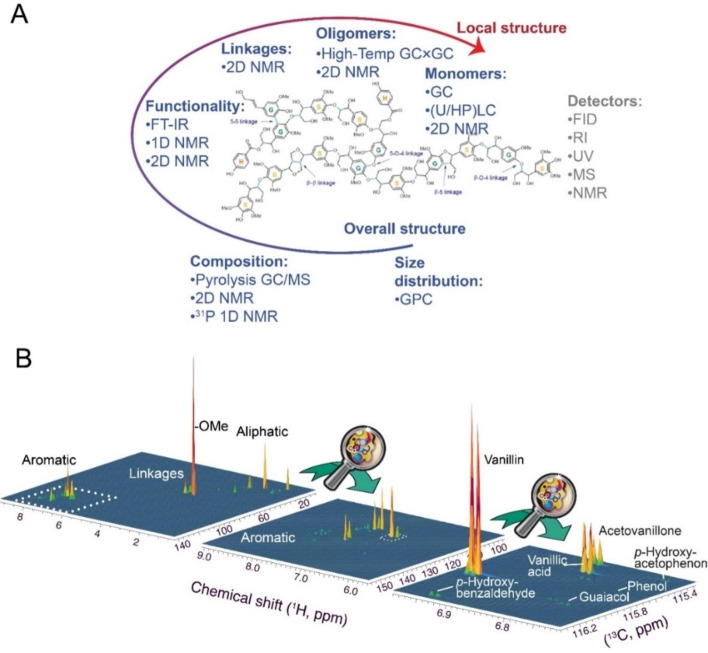
(A) Overview of commonly addressed research questions, methods, and detectors employed in studies of oxidative lignin conversion. The lignin structure is adapted from Ref. [25], under the terms of the Creative Commons Attribution 4.0 International License (http://creativecommons.org/licenses/by/4.0/). (B) Analysis of bio‐oil from oxidative lignin degradation with ^1^H–^13^C HSQC spectroscopy. 2D NMR methods can benefit from the appropriate choice of spectral width in the second dimension and are highly reproducible. The measurement time in the right‐most spectrum (acquired in DMSO‐*d*_6_) was less than 15 min.

Various 2D NMR methods that correlate the chemical shift of two different atoms have been applied to the characterization of lignin, its monomer and linkage composition, and degradation.^[91]^ The most commonly used 2D NMR detection method is ^1^H−^13^C heteronuclear single‐quantum coherence (HSQC), which benefits from the 32‐fold higher sensitivity than in the 1D ^13^C NMR spectrum, while still exploiting the approximately 20‐fold higher chemical shift range in the ^13^C dimension relative to the ^1^H dimension.^[92]^ NMR spectroscopy can contribute to the identification of novel chemicals and is advantageous in the detection of all classes of organic substrate that contain the nucleus or functional group that is detected in the experiments (Figure 8). 2D NMR methods can benefit from the appropriate choice of spectral width in the second dimension, using sparse sampling to accelerate data acquisition, and from the use of high‐field instrument centers for sensitive high‐resolution measurements. Baseline‐separated repeatable measurements can be particularly useful in providing definitive insight into processes run under specific reaction conditions, and the products obtained.^[18]^ While not being intrinsically quantitative, due to site‐specific differences in magnetization transfer and magnetization loss in 2D NMR spectroscopy, various experimental approaches, such as calibration and the use of special pulse sequences, can be used to transform 2D NMR spectroscopy into a quantitative analytical and predictive tool.[^93^, ^94^, ^95^]

Methodological and experimental developments, together with the integration of complementary, multimodal methods of analysis, will be key to the efficient discovery and characterization of pathways that allow the formation of lignin‐sourced phenolic compounds and aliphatic organic acids. The ultimate goal is a comprehensive platform that can characterize the fate of lignin in gas, liquid, and solid fractions, and identify the predictive relationship between the characteristics of lignin and the outcome of processes.

### 3.6 Product recovery in the oxidative valorization of technical lignin

The reaction mixture resulting from oxidative lignin depolymerization generally consists of a complex cocktail containing lignin oligomers, low‐molecular‐weight aromatics, and minor amounts of other secondary non‐phenolic compounds.[^41^, ^96^] The type and amount of each oxidation product in the final mixture depend greatly on the lignin raw material, the processing conditions, and the chemicals added. However, most published studies focus on the specific lignin‐derived aromatic compounds that are of interest. These compounds are usually di‐functional, and include aromatic aldehydes (i. e., vanillin and syringaldehyde), their respective aromatic acids (vanillic and syringic acids), and ketones (acetovanillone and acetosyringone).[^42^, ^43^] The chemical and physical similarities between these aromatic monomers (i. e., their molecular weight, acid dissociation constant, density, and melting point) affect their separation from the oxidized lignin mixture and their consequent purification process.^[97]^

Most of the research studies reported in the literature have focused on obtaining purified fractions of the aromatic compounds of interest. The main sequences of downstream processes used for oxidized lignin mixtures include liquid–liquid extraction (LLE), membrane separation, adsorption, ion exchange, distillation, acidification/precipitation, bisulfitation, supercritical fluid extraction (SFE), and crystallization.[^41^, ^42^, ^97^, ^98^, ^99^, ^100^]

Among the variety of compounds resulting from oxidative lignin depolymerization, vanillin and syringaldehyde usually represent the major products, but still make up only a small fraction of the product mixture. The greatest challenge is to find the most effective method of separating these high‐value aromatic aldehydes in as pure a form as possible from the product mixture, given the diversity of processes available. The two major problems cited in this field are the acidification of the mixture and the removal of the residual lignin in solution.^[43]^ One of the traditional processes for the production of vanillin from waste sulfite pulp liquor involves acidification of the oxidized medium for lignin precipitation, extraction of the aromatics of interest with volatile organic solvents, and further purification.^[42]^ However, this sequence of processes requires high volumes of chemicals and solvents, and the mixture obtained remains difficult to purify. These difficulties compromise the environmental sustainability and economic viability of the process.

Membrane separation is often used to reduce the complexity of the oxidized lignin mixture by separating the high‐molecular‐weight fractions of degraded lignin from the lower‐molecular‐weight species. From a separation point of view, membrane selection depends on the characteristics of the membrane, and a trade‐off is necessary between high selectivity and high recovery.^[101]^ Ultra‐ and nanofiltration membrane sequence^[99]^ and nanofiltration membrane cascades^[101]^ have already been studied and demonstrated to be successful in the separation of fractions with different molecular weights, overcoming some limitations such as low fluxes, rejection, and fouling. However, membrane separation by itself is not sufficient for the effective separation of fractions rich in the desired aromatic compounds, and it must be combined with other techniques.

Adsorption allows the separation of selected compounds in the dilute solutions resulting from ultrafiltration of oxidatively depolymerized lignin mixtures. This process is based on the polarity of the compounds and is attractive for its relative simplicity of design, operation, and scale‐up, high capacity and favorable rate, insensitivity to toxic substances, ease of regeneration, and fairly low cost.[^99^, ^102^] Additionally, adsorption avoids the use of toxic solvents and minimizes degradation. Despite these advantages, other processes must be combined to obtain a higher degree of purification of the phenolic monomers.

**Figure 9:**
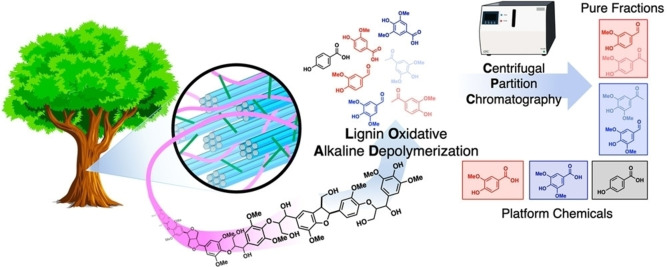
Illustration of centrifugal partition chromatography as an effective means of obtaining pure fractions of aromatic chemicals from oxidative lignin depolymerization. Reproduced from Ref. [103], under the terms of the Attribution‐NonCommercial‐NoDerivatives 4.0 International License (https://creativecommons.org/licenses/by‐nc‐nd/4.0/).

In a recent innovative study by Stahl and co‐workers,^[103]^ centrifugal partition chromatography (CPC) was demonstrated to be an effective method of isolating individual aromatics from an oxidatively depolymerized poplar‐derived lignin mixture (Figure 9). The LLE method involves two stages of ascending‐mode extraction using two different solvent systems, that is, pentane/ethyl acetate/methanol/water (2 : 3 : 2 : 3) and dichloromethane/methanol/water (10 : 6 : 4). Vanillin, syringic acid, and oligomers were recovered in the first stage, while syringaldehyde, vanillic acid, and 4‐HBA were recovered in the second stage. Since the recovery of several aromatics typically requires several unit operations, CPC has considerable potential as a scalable separation method for oxidatively depolymerized lignin products.

The final purification process represents a significant challenge due to the presence of phenolic side‐products with chemical and physical properties similar to those of the aromatic aldehydes of interest. For instance, the main side‐products obtained from the oxidative depolymerization of softwood lignin consist mainly of vanillin‐related species, such as *o*‐vanillin (2‐hydroxy‐3‐methoxybenzaldehyde), 5‐formyl vanillin (4‐hydroxy‐5‐methoxyisophthalaldehyde), vanillic acid, and acetovanillone.^[41]^ Crystallization and distillation could provide a final product with the desired high purity; however, both processes should be applied in the final sequence of separation and purification when the compounds of interest are present at low volumes and high concentrations. Crystallization usually requires several steps, some of which involve derivatization, in order to achieve high‐purity vanillin and/or syringaldehyde.^[104]^ On the other hand, distillation requires high temperatures, which could cause degradation of the compounds of interest. Vacuum distillation, fractional distillation, carrier steam distillation, and azeotropic distillation have been described previously to obtain a purified vanillin stream from depolymerized lignin mixtures.[^98^, ^105^, ^106^] However, other separation processes, such as LLE, SFE, or crystallization, are often required after distillation.

In light of possible methods for the separation and purification of lignin‐derived products obtained via the oxidative depolymerization of lignin, it can be inferred that a sequence of separation and purification processes will be necessary to achieve a sustainable process. The development of such a sequence should focus on recovering vanillin and/or syringaldehyde, in as pure a form as possible, from the other low‐molecular‐weight aromatic compounds present in the oxidized lignin mixture. The recovery of other valuable compounds associated with the product mixture resulting from oxidative lignin depolymerization, such as aromatic acids and ketones, remains an important subject for future research. However, it is important to bear in mind that not only the efficiency and effectiveness of the separation and purification of the aromatics, but also the economic and environmental viability must be considered in future industrial applications.

## 4 Conclusions and Perspectives

The need for renewable precursors in the production of chemicals, fuels, and materials, together with the large and underutilized lignin side‐stream from the pulp and paper industry, motivate efforts to valorize technical lignin. A win–win situation could be reached by addressing the bottlenecks in pulp production in combination with the extraction of lignin from black liquor. Economic advantages could be achieved, while simultaneously diversifying the product portfolio from lignin, for example, by recovering aromatic monomers produced via depolymerization. Although lignin depolymerization is not a new area of research, several challenges remain to be overcome before a sustainable and economically viable process can be achieved.

Catalytic oxidative depolymerization is a promising route for lignin conversion to value‐added products due to its mild reaction conditions and the possibility of using inexpensive and green oxidants such as air or oxygen. Heterogeneous catalysts have the advantages of easier separation and regeneration of the catalyst than homogeneous systems. Although numerous studies were dedicated to this approach, the majority of these studies employed lignin model compounds as substrates, and the findings often cannot be extrapolated to real lignin or native lignin. The starting material strongly affects the results of oxidative lignin depolymerization, and can lead to operational problems, such as catalyst deactivation. Attention should be directed to technical lignin in order to achieve efficient catalytic oxidative depolymerization that can be applied industrially for the utilization of the aromatic feedstock provided by lignin side‐streams.

One of the significant challenges highlighted in this Review is the stability and recyclability of the heterogeneous catalysts used in oxidative lignin depolymerization. A high number of catalyst systems exhibited metal leaching or deactivation resulting from the deposition of organic material or other impurities. Additionally, the detailed mechanisms behind catalytic deactivation and regeneration have remained unclear. Therefore, the emphasis in future research should be on developing stable and robust catalytic systems for oxidative lignin conversion.

The complexity of lignin and lignin‐derived product mixtures still limits the insight that can be obtained from individual analytical techniques. The development of analytical methods will be needed to obtain comprehensive information on suitable pathways and products. Furthermore, little attention has been paid to the separation of depolymerized lignin products,^[107]^ whereas developing an efficient separation process should go hand in hand with depolymerization in order to improve the upstream and downstream processes for oxidative lignin valorization. The recovery of aromatic products resulting from oxidative lignin depolymerization will involve a complex sequence of separation and purification processes, since the reaction mixture usually contains other products with similar physical and chemical properties. Most processes for the separation and purification of the aromatic monomers of interest, such as vanillin and syringaldehyde, are very labor‐ and energy‐demanding, and involve environmentally harmful solvents, and high material losses. Developing an efficient and environmentally friendly separation process is thus one of the most important tasks towards the industrial application of lignin‐derived aromatics.

## Conflict of interest

The authors declare no conflict of interest.

5

## Biographical Information

*Omar Y. Abdelaziz obtained his Bachelor's (2013) and Master's (2015) degrees from Cairo University, Egypt, and his Ph.D. (2021) in Chemical Engineering from Lund University, Sweden. His Ph.D. research focused on developing technologies for the thermochemical depolymerization of technical lignin into value‐added chemicals. He is currently a Postdoctoral Researcher in the group of Prof. Christian P. Hulteberg at Lund University, where he continues to explore novel routes for lignin valorization. He is also a co‐founder of a start‐up company dealing in lignin‐based batteries*.

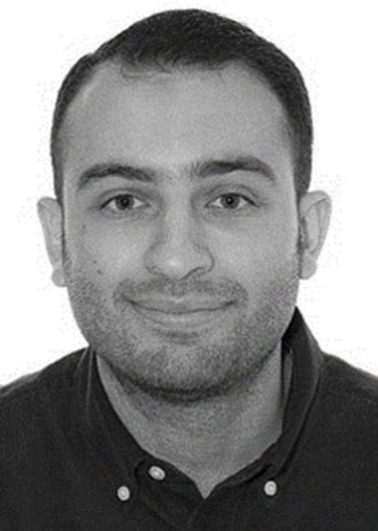

## Biographical Information

*Anders Riisager is a Professor at the Department of Chemistry, Centre for Catalysis and Sustainable Chemistry, Technical University of Denmark (DTU). His background is in inorganic materials chemistry and catalysis. Prior to his current position, he has completed four years of postdoctoral research at RWTH‐Aachen/University of Erlangen‐Nuremberg, Germany and been appointed as Associate Professor at DTU. His major scientific focus is on the development of sustainable chemistry with catalysis and renewables as well as chemical technology with ionic liquids. Several of the developed technologies are commercialized to industry and he is co‐founder of two companies focusing on biomass valorization technology*.

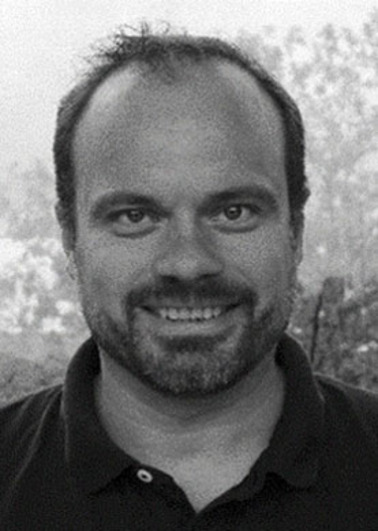

## Data Availability

Data sharing is not applicable to this article as no new data were created or analyzed in this study.
